# Multi-part segmentation for porcine offal inspection with auto-context and adaptive atlases

**DOI:** 10.1016/j.patrec.2018.07.031

**Published:** 2018-09-01

**Authors:** Stephen McKenna, Telmo Amaral, Thomas Plötz, Ilias Kyriazakis

**Affiliations:** a CVIP, School of Science and Engineering, University of Dundee, Dundee DD1 4HN, UK; b Open Lab, School of Computer Science, Newcastle University, Newcastle upon Tyne NE1 7RU, UK; c Agriculture, School of Natural and Environmental Sciences, Newcastle University, Newcastle upon Tyne NE1 7RU, UK; d School of Interactive Computing, College of Computing, Georgia Institute of Technology, Atlanta, GA, USA

**Keywords:** Multi-class segmentation, Auto-context, Atlas-based segmentation, Automated inspection, 41A05, 41A10, 65D05, 65D17

## Abstract

Extensions to auto-context segmentation are proposed and applied to segmentation of multiple organs in porcine offal as a component of an envisaged system for post-mortem inspection at abbatoir. In common with multi-part segmentation of many biological objects, challenges include variations in configuration, orientation, shape, and appearance, as well as inter-part occlusion and missing parts. Auto-context uses context information about inferred class labels and can be effective in such settings. Whereas auto-context uses a fixed prior atlas, we describe an adaptive atlas method better suited to represent the multimodal distribution of segmentation maps. We also design integral context features to enhance context representation. These methods are evaluated on a dataset captured at abbatoir and compared to a method based on conditional random fields. Results demonstrate the appropriateness of auto-context and the beneficial effects of the proposed extensions for this application.

## Highlights

• Automatic segmentation of multiple organs using auto-context.
• Auto-context extended using integral context features and adaptive atlases.
• Evaluation on image dataset of porcine offal.
• Proposed extensions to auto-context improved segmentation.

## 1 Introduction

Segmentation of non-rigid biological objects into their constituent parts presents various challenges. Here we address a segmentation task in which parts are organs in body images captured at abbatoir. This constitutes one stage in an envisaged on-site system for screening of pathologies; these are characteristically organ-specific. The spatial arrangement of organs in an image is only weakly constrained and their shape is variable. Furthermore their appearance changes due to factors including cause of pathology, surface contaminants, and specular reflections. There can be limited control over orientation, severe occlusions between parts, and parts may be missing altogether. In this paper we describe adaptations to the auto-context (AC) segmentation algorithm to address such a task. We apply these to segment heart, lungs, diaphragm and liver in porcine offal. The groups of inter-connected organs are called *plucks*, examples of which are shown in Figs. 2 and 3.

Auto-context [3] is an iterative technique that combines *contextual* classification information with local image features. AC is relatively flexible and easy to implement, and has been applied to various biomedical imaging problems  [3], [4]. The context features used by AC to inform class label inference at a pixel location are posterior class probabilities produced by the previous iteration. These probabilty values are typically sampled at a fixed set of locations relative to the pixel in question. Additionally we design *integral context* features obtained by summing probability values over sets of locations. In the application considered here we argue that sums over rows and sums over the entire foreground are appropriate.

One attractive feature of AC is that a prior *atlas* can be used as a source of contextual data for the initial iteration. Such an atlas can be obtained by averaging rigidly registered manual segmentation maps. However, a single averaged map does not provide a good representation of the multi-modal map distribution that arises as a result of the variations mentioned above, such as occlusions and missing parts. We describe weighted atlas auto-context (WAAC), a method that adapts an atlas representation to be relevant to the current image. This improved atlas is used at the next iteration as an additional source of information together with the label probability maps.

In this paper we combine integrated context and WAAC into one system, extending work reported in conference papers on integral context [1] and WAAC [2]. We report a direct comparison of all of these methods applied to segmentation of multiple organs in pig offal, and we also compare with a conditional random field (CRF) method. We evaluate performance in terms of Dice coefficient distributions, pixel-wise classification and quadratic scores.

## 2 Background

Post-mortem inspection is an important means of ensuring the safety and quality of meat products, enabling the detection of public health hazards and pathologies, and providing useful feedback to farmers. There are moves towards visual-only inspection of pig carcasses and offal without palpation, in order to minimise risk of cross contamination [5], [6]. This along with the potential to detect a greater number of pathologies with improved reproducibility than currently possible with manual inspection [7] motivates development of automated visual inspection. Reliable segmentation of organs would constitute an important step towards this goal. In this context even modest improvements in organ segmentation could be significant as regions assigned to the wrong organ may ultimately lead to missed or falsely detected pathologies.

Applications to meat production deal mostly with estimation of proportions of muscle, fat and bone either *in vivo* and post-mortem, sometimes involving segmentation of organs without distinguishing them individually [8], [9]. Tao et al. [10] segmented poultry spleen from surrounding viscera as an aid to detection of splenomegaly. Jørgensen et al. [11] segmented gallbladders in chicken livers from images acquired at two visible wavelengths. Stommel et al. [12] envisaged a system for robotic sorting of ovine offal that would involve recognition of multiple organs.

Most literature on segmentation of multiple organs deals with human abdominal organs in CT or MR imaging through techniques including level set optimisation [13], statistical shape models [14], and atlas-based methods [15], [16].

Segmentation methods that incorporate spatial context information include those combining inference algorithms based on belief propagation (BP) [17] with models like conditional random fields (CRFs) [18]. Disadvantages common to many such techniques that aim to capture context information include their reliance on fixed spatial configurations with confined neighbourhood relations and complex training procedures.

There is extensive literature dealing with the construction of unbiased atlases for multi-modal data, especially in brain magnetic resonance (MR) image analysis, as in the work of Blezek and Miller [19] and Zikic et al. [20]. Some related work makes use of AC. Kim et al. [21], for example, employed an approach similar to that of Zikic et al. [20], training multiple models, each based on an individual annotated image, so that the probability map of a new image was obtained by averaging maps predicted by individual models. Zhang et al. [22] proposed a hierarchy of AC models whose bottom level is similar to the set of models used by Zikic et al. [20] and Kim et al. [21]. Given a new image, only the best models in the hierarchy are selected to contribute to the final probability map. Model training via these techniques can be computationally expensive.

## 3 Methods

### 3.1 Auto-context (AC)

We perform segmentation using methods built around the auto-context (AC) algorithm of Tu and Bai [3]. AC learns to map an input image to a multi-class segmentation map consisting of posterior probabilities over class labels. It iteratively refines the segmentation map by using the label probabilities in a given iteration as a source of contextual data for the following iteration. Label probabilities at a set of locations relative to the location to be classified are concatenated with local image features to form a combined feature vector for training the next classifier.

Let *S* be a set of *m* training images *X_j_* together with their label maps *Y_j_*, i.e. $S=\{\left(Y_{j},X_{j}\right),j=1..m\}$. At each iteration *t* we want to train a classifier that outputs the probability distribution $p_{ji}^{\left(t\right)}$ over labels $y_{\mathrm{ji}}\in \left\{1..K\right\}$ for pixel *i* in image *X_j_*, given the image patch *X_j_*(*N_i_*) from which local features are computed, and label probability map $P_{j}^{\left(t-1\right)}\left(i\right)$ (see Eq. (1)).

(1) $$p_{ji}^{\left(t\right)}=p\left(y_{ji}|X_{j}\left(N_{i}\right),\;P_{j}^{\left(t-1\right)}\left(i\right)\right)$$

In *X_j_*(*N_i_*), *N_i_* denotes all pixels in the image patch, and $P_{j}^{\left(t-1\right)}\left(i\right)$ is map $P_{j}^{\left(t-1\right)}$ output for image *X_j_* at the previous iteration t−1, but now centred on pixel *i*.

AC produces a sequence of classifiers, one per iteration. Before the first iteration, all probability maps $P_{j}^{\left(0\right)}$ can be initialised using a prior atlas *Q*^(0)^, obtained by averaging *m* training label maps:

(2) $$Q^{\left(0\right)}=\frac{1}{m}\sum_{j}Y_{j}.$$

At each iteration, given pixel *i* in image *X_j_*, the actual feature vector input to the classifier is composed of local image features extracted from patch *X_j_*(*N_i_*) concatenated with context features extracted from the re-centered label probability map $P_{j}^{\left(t-1\right)}\left(i\right)$. Context features are the probabilities extracted from selected locations on map $P_{j}^{\left(t-1\right)}\left(i\right),$ including the central location that corresponds to the current image pixel *i*. Selected locations are typically defined by a sparse star-shaped “stencil”.

In our implementation of AC, context probabilities for a location are extracted at 90 surrounding stencil points as well as at the location itself. At the first iteration, context consists of the 5 class label probabilities provided by the prior atlas at each of the 91 associated context points; at subsequent iterations, it consists of the label probabilities output by the classifier at the previous iteration, at the same context points. This gives 91  ×  5=455 context features per image point. We use multi-layer perceptron classifiers (MLPs); these can be trained to directly estimate posterior probability distributions over the class labels.

### 3.2 Integral context (IC)

Context data can be enhanced by including integral features, i.e. sums of class label probabilities. We augment the context features described above with two types of integral context features suitable for our application.

The relative positions of organs along the vertical direction vary little from image to image, given that each pluck hangs from a hook and the part of the pluck that is attached to the hook is very consistent across plucks. Thus, given a point on an image, class probabilities averaged over the *row* to which the point belongs provide the classifier on the next iteration with useful information as to which organs are likely to occur at that particular height. For example, a row containing heart is likely to contain also lungs, but very unlikely to contain liver.

In contrast, relative positions of organs along the horizontal direction vary considerably from image to image, given lack of control over the orientation of the pluck around the vertical axis. The heart, in particular, is sometimes fully occluded. Nevertheless, organs are fairly consistent in volume from pig to pig. Thus, class probabilities averaged over the *whole* image reflect the proportions of the pluck covered by each visible organ, and provide the next classifier with useful information on which organs are likely to be visible and how visible they are. For example, a small proportion of visible diaphragm is consistent with a hidden heart and a large proportion of lung.

We use IC to refer to methods in which these integral context features are included, i.e. the sum of the label probabilities in the row and the sum of label probabilities in the entire image.

### 3.3 Weighted atlas auto-context (WAAC)

At the end of each training iteration *t*, for each image *X_j_* we can select the training annotations *Y_k_* closest to probability map $P_{j}^{\left(t\right)}$ output by the classifier, assign a weight to each selected annotation, and combine them to obtain a weighted atlas $Q_{j}^{\left(t\right)},$

(3) $$Q_{j}^{\left(t\right)}=\frac{1}{\sum_{k\neq j}s_{kj}^{\left(t\right)}w_{kj}^{\left(t\right)}}\sum_{k\neq j}s_{kj}^{\left(t\right)}w_{kj}^{\left(t\right)}Y_{k}.$$

In Eq. (3), weight $w_{kj}^{\left(t\right)}$ is a measure of similarity between label map *Y_k_* and probability map $P_{j}^{\left(t\right)},$ and $s_{kj}^{\left(t\right)}$ is a selection variable defined as:

(4) $$\begin{array}{c} s_{\mathrm{kj}}^{\left(t\right)}=\left\{ \begin{array}{cc} 1 & \text{if}k\in K_{j}^{\left(t\right)}\\ 0 & \text{otherwise} \end{array}\right. \end{array}$$

where $K_{j}^{\left(t\right)}$ denotes the set of indices of the *m_w_* largest weights in $\left\{w_{\mathrm{lj}}^{\left(t\right)}|l=1..m\right\}$. We refer to this method as WAAC. For the similarity measure $w_{kj}^{\left(t\right)}$ we chose to use the mean class *F*_1_-score between label map *Y_k_* and probability map $P_{j}^{\left(t\right)}$. The *F*_1_-score for a given class is defined as the harmonic mean of precision *p* and recall *r* for that class, that is, 2pr/(p+r). For each class, a high precision means that most of the predicted region is contained in the true region, whereas a high recall means that the predicted region contains most of the true region. Thus, a high *F*_1_-score will normally correspond to predicted regions whose boundaries closely match those of the true regions. This is particularly important when segmenting multiple adjacent parts belonging to different classes.

**Fig. 1:**
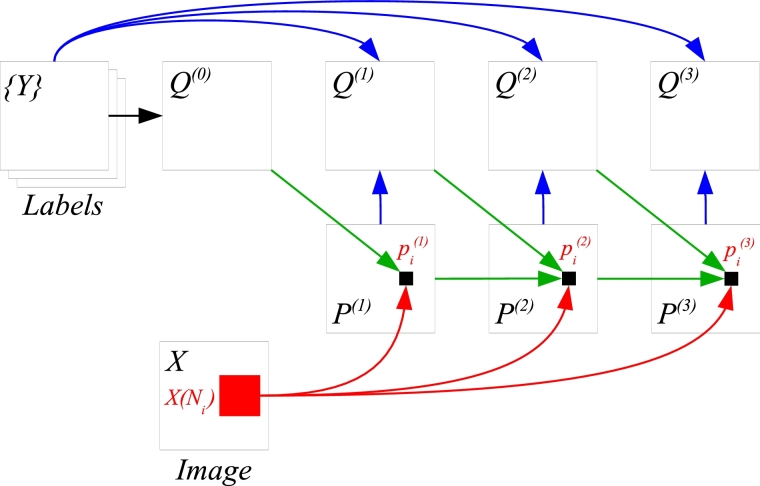
Use of a WAAC model (shown for 3 iterations) on a test image *X*. Red and green arrows correspond to the use of a classifier at each iteration to classify a pixel (represented by the small black square), whereas blue arrows correspond to use of Eq. (3) at each iteration to obtain a weighted atlas. The large red square represents an image patch centred on the pixel being classified, used for the extraction of local appearance features. (For interpretation of the references to colour in this figure legend, the reader is referred to the web version of this article.)

Algorithm 1 summarises WAAC training; parts that differ from conventional AC are highlighted. At the start of a WAAC training iteration, features are extracted from the weighted atlas computed at the end of the previous iteration, in addition to conventional AC features. The first iteration can in principle be run as conventional AC, to avoid providing duplicate features to the classifier. (Note that, for any given image *X_j_*, both $P_{j}^{\left(0\right)}$ and $Q_{j}^{\left(0\right)}$ would merely be copies of prior atlas *Q*^(0)^.) The schematic in Fig. 1 shows use of a trained WAAC model on a test image.

**Algorithm 1:**
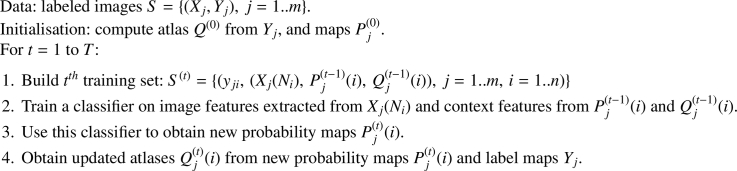
WAAC training. Highlights are WAAC-specific.

**Fig. 2:**
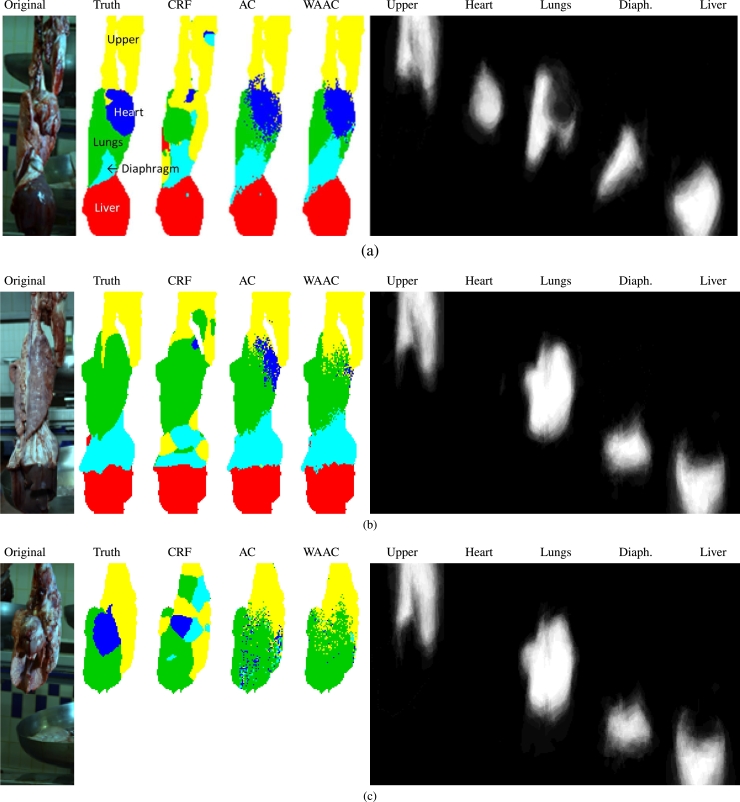
Three examples showing input image, ground-truth and output obtained using CRF, AC, and WAAC. The organ-specific components of the weighted atlas obtained at the final iteration of WAAC are also shown. The upper, heart, lungs, diaphragm and liver classes are shown in yellow, blue, green, cyan and red, respectively. The upper class denotes the portion of the pluck located above the heart and lungs usually consisting of the trachea and tongue. In (b) the heart is occluded, whereas in (c) the liver is missing from the pluck. (Best viewed in colour.). (For interpretation of the references to colour in this figure legend, the reader is referred to the web version of this article.)

**Fig. 3:**
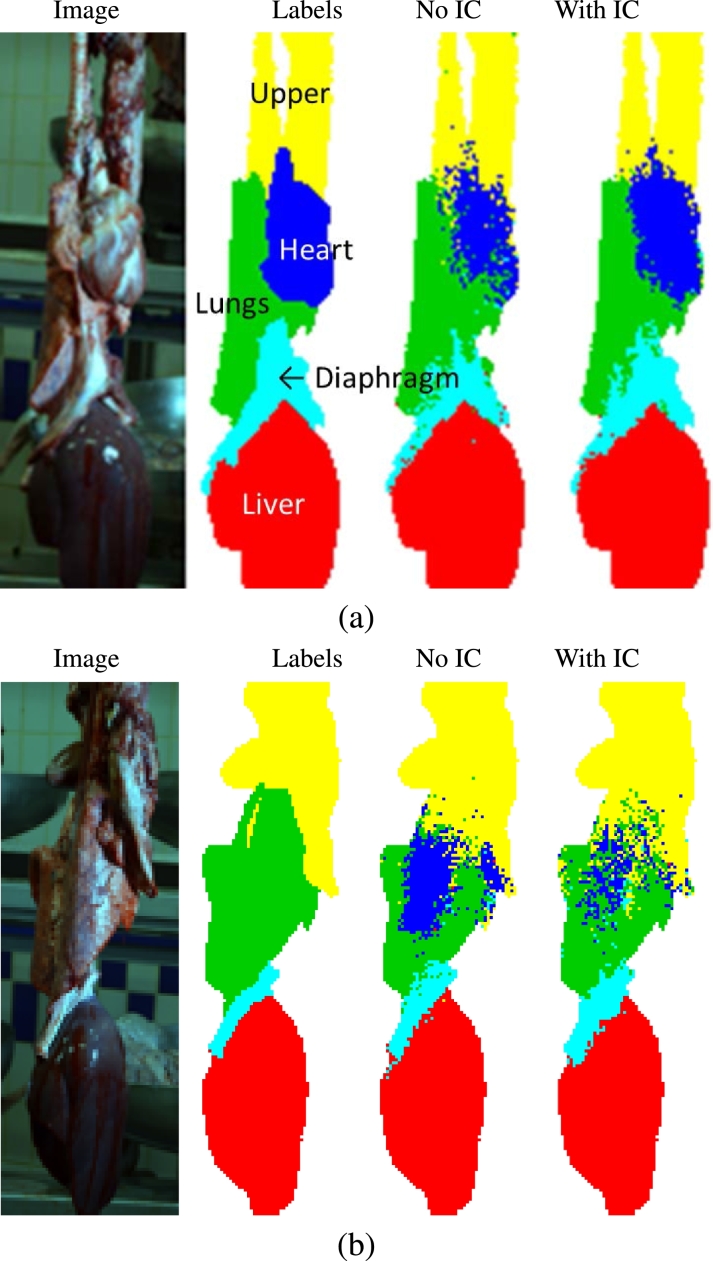
Two examples showing input image, ground-truth labels, and output obtained using AC with and without use of integral context (IC) features. (Best viewed in colour.)

WAAC uses the same number and spatial arrangement of context points as AC; in other words, there is no additional spatial context. At each iteration, WAAC combines information from two sources that are very different in nature: the probability maps output by the classifier (as in AC); and a weighted atlas obtained from the ground-truth component of training data.

## 4 Dataset

The dataset consisted of 350 annotated colour images of plucks in an abattoir production line. The images were acquired under LED illumination using a single-lens, tripod-mounted, reflex digital camera. Each image had a resolution of 3646  ×  1256 pixels. Four organ classes were manually annotated in each image: the *heart, lungs, diaphragm* and *liver*. A fifth class, *upper*, was used to mark the portion of the pluck located above the heart and lungs usually consisting of the trachea and tongue. Fig. 2 shows some pluck images along with annotations showing the regions occupied by each organ class.

## 5 Validation and implementation details

The 350 available images were randomly divided into 10 subsets of 35 images. Those subsets were used to carry out 10-fold cross validation experiments comparing the performance of CRFs, conventional AC, and the proposed WAAC method.

We used local appearance features based on a multi-level Haar wavelet decomposition [23]. Each image was converted to the CIELUV colour space [24]. For each component (L*, u*, and v*), the approximation wavelet coefficients, as well as the horizontal, vertical, and diagonal squared detail coefficients, were obtained at three levels of decomposition. This resulted in 36 feature maps (3 image components  ×  4 wavelet coefficients  ×  3 levels of decomposition), all rescaled to match the original dimensions of the image. We then sub-sampled each feature map and each label map by a factor of 20 along both dimensions. This resulted in 180  ×  60 points per map, which was found to provide sufficient detail for our purposes.

Each pluck had already been segmented from the background using a relatively trivial segmentation step based on focus and hue information. Auto-context methods were trained at foreground locations on a rectilinear grid. There were approximately 2 m such locations in the dataset (5.7k per image). At each cross-validation fold, a balanced training set was obtained by stratified sampling of 8000 locations (1600 per class). Each training pair consisted of a vector of local and context features and the corresponding class label. When training auto-context methods, performance tended to saturate after five iterations [2]. Therefore we set T=5. When training WAAC models, 32 annotations (10% of a fold’s training pool) were used to compute each weighted atlas ($m_{w}=32$). As reported previously, pixel classification accuracy was stable when *m_w_* was varied over an order of magnitude [2].

MLPs had a softmax output and a hidden layer of 20 neurons with logistic activation functions. They were trained with an *L*_2_-regularised cross-entropy loss using scaled conjugate gradients optimisation in the Netlab implementation [25]. The CRF model used for comparison [26] was implemented with the toolbox for Matlab / C++ made available by Domke [27]. A 180 × 60 pairwise 4-connected grid was created to match the dimensions of our feature and label maps. CRF models were trained for five iterations of tree-reweighted belief propagation to fit the clique logistic loss, using a truncated fitting strategy.

## 6 Results

### 6.1 Qualitative results

We first discuss some example results obtained using AC, WAAC and CRF. Fig. 2 shows pixel labellings obtained, by assigning labels with highest probabilities, from three pluck images. The CRF method produced smooth inter-organ boundaries but made gross labeling errors; some regions were labeled in implausible locations, for example small regions of heart and diaphragm near the top of the upper segment in Fig. 2(a), and upper regions below the lungs in Fig. 2(b). When the highest probabilistic outputs from AC and WAAC were used to obtain class labels, high frequency class transitions occured. The use of the adaptive atlas in WAAC tended to improve spatial coherence compared to AC. Note that these results are presented without any attempt to apply post-processing to smooth the labellings.

The organ-specific atlas components obtained at the final iteration of WAAC are also shown in Fig. 2. The atlas has clearly adapted differently to the different spatial configurations in the input images. In Fig. 2(b) it has adapted to exclude the heart which is indeed occluded in that example. Fig. 2(c) shows a difficult example for which all the methods failed. In this unusual case the liver, which is nearly always present, is missing entirely. This eventuality was not well represented in the training data. The methods did manage to exclude liver from their results but the mismatch resulted in poor localisation of other organs in the image.

For a further two test images, Fig. 3 shows results obtained without and with integral context. Note that a simple denoising post-processing step would have improved the quality of segmentation results, but we left that step out to more clearly show the effect of adding integral context. The importance of integral features is most visible in cases like that of Fig. 3(a), in which standard (stencil based) context was not enough to yield a confident segmentation of the heart. Fig. 3(b) illustrates the reverse situation, where integral features helped to dissipate a mistakenly segmented heart. In this case, the integral features representing class probabilities averaged over the whole image will have reflected the small area occupied by the diaphragm and large area covered by the liver, thus helping to identify a pluck whose dorsal aspect faced the camera, hiding the heart.

### 6.2 Organ-level evaluation

**Fig. 4:**
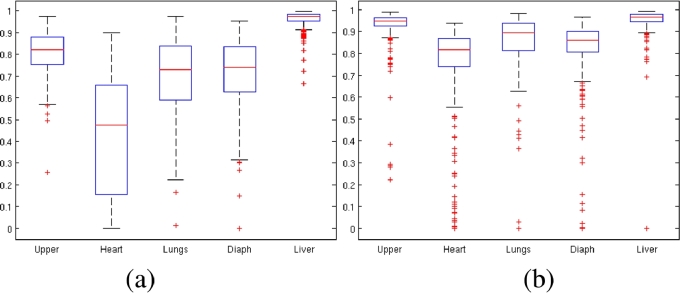
Dice scores for (a) the conditional random field model (CRF), (b) weighted atlas auto-context with integral context features (WAAC+IC).

For each test image, a Dice score was computed for each organ using the formula 2|X∩Y|/(|X|+|Y|), where |*X*| is the number of pixels that belong to the organ in the ground-truth image, |*Y*| is the number of pixels that belong to the organ in the predicted image, and |*X* ∩ *Y*| is the number of pixels that belong to the organ both in the ground-truth image and in the predicted image. Any scores where the organ was not present in the ground truth image were left out. Table 1 gives median Dice scores for each of the five methods for each of the five object classes. The final row gives the average of the five class-specific values. Box and whisker plots in Fig. 4 show how Dice scores were distributed in the case of CRF and WAAC+IC. The auto-context segmentation methods outperformed the CRF method. Integral context features and the use of a weighted atlas both had a beneficial effect on Dice coefficients.

**Table 1:** Median Dice coefficients.

	CRF	AC	AC+IC	WAAC	WAAC+IC
Upper	0.820	0.936	0.946	0.944	**0.947**
Heart	0.475	0.792	0.810	0.812	**0.816**
Lungs	0.730	0.868	0.881	0.883	**0.894**
Diaph.	0.740	0.849	0.857	0.859	**0.862**
Liver	**0.973**	0.962	0.968	0.965	0.966
Avg.	0.748	0.875	0.888	0.886	**0.891**

### 6.3 Pixel-level evaluation

Table 2 gives confusion matrices obtained from CRF and WAAC+IC when used to perform classification at the pixel level by assigning each pixel to the class with the highest probability. After its 5th iteration, the CRF method performed at a similar level to AC before context iterations.

**Table 2:** Confusion matrices: (a) CRF (b) WAAC+IC.

(a)					
Ground truth	Predicted				
	Upper	Heart	Lungs	Diaph.	Liver
Upper	401,130	11,019	58,100	15,322	484
Heart	25,864	81,715	58,974	14,565	1340
Lungs	58,549	26,213	421,258	23,428	4884
Diaph.	19,761	8664	39,894	191,760	15,964
Liver	1285	1086	5476	8160	523,815
(b)					
Ground truth	Predicted				
	Upper	Heart	Lungs	Diaph.	Liver

Upper	445,393	22,387	15,842	1964	469
Heart	10,471	158,131	11,853	1980	23
Lungs	18,757	32,910	454,525	27,552	588
Diaph.	1634	3460	12,491	244,946	13,512
Liver	1212	2787	886	25,091	509,846

The largest improvement apparent in the WAAC+IC result was observed for the heart. Being relatively small, the heart is the organ whose two-dimensional projection on each image is most affected by the orientation of the pluck around its vertical axis: it can be fully visible near the centre, partially or fully visible on either side of the pluck, or completely hidden. Thus, it is not surprising that the ability of WAAC to deal with multi-modality had a larger impact on the segmentation associated with this organ. Integral context features also helped to deal with the unpredictability of the heart’s presence and position in the image.

Various proper scoring rules can be used to measure the accuracy of probabilistic predictions [28]. We computed the quadratic score $Q\left(r,i\right)=2r_{i}-r\cdot r$ where *r_i_* is the probability assigned to the ground-truth class. For each image, the average quadratic score was computed. Table 3 reports the median of this; auto-context methods again outperformed the CRF. Integral context features and the use of a weighted atlas both had a beneficial effect on pixel-level performance.

**Table 3:** Median of images’ quadratic scores.

CRF	AC	AC+IC	WAAC	WAAC+IC
0.732	0.829	0.845	0.845	**0.854**

### 6.4 Computational cost

Execution times were measured running on a regular desktop machine, using only the CPU (an Intel Core i7-870). Processing an image at test time was dominated by feature extraction which took 7.2*s*. One iteration of AC took 0.14*s* whereas an iteration of WAAC took 0.73*s* due to the extra computation needed to compute the weighted atlas. The feature extraction and atlas computation routines were implemented in Matlab. The computation of weighted atlases would be easily adapted for faster execution on a GPU.

## 7 Conclusion

We introduced the problem of multiple organ segmentation at abattoir and proposed solutions based on an auto-context approach. Specifically, we described two modifications of auto-context for multi-part segmentation. Firstly, the stencil-based context features were augmented with integral features. Secondly, a weighted atlas was iteratively adapted and made available for the extraction of features to complement those used in the conventional approach. Experiments on the task of segmenting multiple organs in images of pig offal acquired at abattoir demonstrated the effectiveness of this approach. It outperformed an alternative CRF method and was able to deal with parts whose spatial arrangement, appearance and form varied widely across images, most noticeably when segmenting the heart which was often severely occluded. Taking advantage of the iterative nature of AC, WAAC is able to identify the training label maps that are most relevant for a given test image and use that knowledge to steer the segmentation process, thus helping to avoid the erroneous localisation of parts within conflicting contexts. Future work could include the computation of weighted atlases in a class-wise fashion, the use of alternative similarity measures in the computation of the atlases, and the use of other types of classifier within the WAAC algorithm which is not restricted to MLPs.

We used auto-context to obtain a sequence of relatively shallow classifiers incorporating label context to achieve semantic segmentation of organs. In recent years, deep neural networks have been designed for semantic segmentation, achieving impressive results in a range of applications albeit on datasets with greater numbers of annotated images [29], [30], [31]. It will be interesting to compare this approach on our inspection task in future work with more annotated images.

The segmentation task evaluated here constitutes a component in an envisaged automated post-mortem inspection application. We describe elsewhere a method for detection of porcine pathologies (specifically pericarditis and liver milk spots) in masked images of pre-segmented organs [32]. This could be integrated with the segmentation methods described in this paper. These methods should also be applicable to other problems involving the segmentation of non-rigid objects into their constituent parts, such as anatomical structures in medical images of various modalities, or sub-cellular compartments in microscopy images.
